# Distribution of lip‐seal strength and its relation to oral motor functions

**DOI:** 10.1002/cre2.440

**Published:** 2021-05-07

**Authors:** Yoshihiro Kugimiya, Takeshi Oki, Midori Ohta, Masahiro Ryu, Kenichiro Kobayashi, Kaoru Sakurai, Takayuki Ueda

**Affiliations:** ^1^ Department of Removable Prosthodontics and Gerodontology Tokyo Dental College Tokyo Japan

**Keywords:** lip pursing, lip‐closing force, lip‐closing strength, lip‐seal strength

## Abstract

**Objectives:**

Lip‐seal strength, which represents the muscle strength of the lips, appears to chiefly contribute to mastication and pronunciation. However, the functional characteristics of lip‐seal strength in adults are still undefined. The present study aimed to understand not only the distribution of lip‐seal strength in adult men and women but also the effect of age on this strength and identify oral motor functions correlated with lip‐seal strength.

**Materials and methods:**

The subjects included 339 participants (men: 170, age 39.2 ± 18.2 years; women: 169, age 43.1 ± 19.7 years). Oral motor function was evaluated for lip‐seal strength, oral diadochokinesis (ODK), tongue pressure, occlusal force, and masticatory performance. Statistical analyses included the Shapiro–Wilk, Mann–Whitney *U*, and Jonckheere–Terpstra tests, in addition to the Spearman's correlation analysis and curvilinear regression analysis.

**Results:**

Lip‐seal strength did not have a normal distribution (*p* < 0.001). The mean ± standard deviation and median (first quartile, third quartile) of lip‐seal strength were 11.2 ± 3.4 and 10.9 (8.7, 13.2)N for the whole sample, 12.3 ± 3.4 and 11.9 (9.4, 14.4)N for men, and 10.2 ± 3.0 and 9.9 (8.0, 12.0)N for women. A significant difference was observed in lip‐seal strength between men and women (*p* < 0.001). Oral motor functions showed a marked correlation with lip‐seal strength, including tongue pressure, occlusal force, and masticatory performance and ODK (/pa/ and /ta/), tongue pressure, and masticatory ability in men and women, respectively. In women, lip‐seal strength declined with increase in age.

**Conclusions:**

Lip‐seal strength was non‐normally distributed in both men and women, and lip‐seal strength was affected by age only in women. Lip‐seal strength and multiple oral motor functions were significantly correlated. Because the indicators of perioral muscle strength and performance were correlated with lip‐seal strength, lip‐seal strength may also partially reflect the condition of the perioral muscles.

## 1 INTRODUCTION

Motor function of the lips is an oral motor activity (Baum & Bodner, 1983; Kikutani et al., 2009; Schimmel, Ono, et al., 2017; Tallgren & Tryde, 1992). The lips are composed of multiple muscles and perform complex movements through their linkage (van Lieshout, 2015). The lips constitute an articulatory organ and an important communication tool in expressing emotion. Furthermore, studies have reported that diverse movements of the lips are related not only to multiple oral motor functions, such as chewing (Ingervall, 1978; Schimmel, Ono, et al., 2017) and swallowing (Hagg & Anniko, 2010), but also to activities of daily living (Miura et al., 2008) and cognitive function (Miura et al., 2008). For maintaining the overall oral motor function and smooth communication, accurately identifying and responding to a decline in the motor function of the lips as early as possible is essential.

The motor function of the lips is evaluated by visual judgment of tension and movement of the lips (Baum & Bodner, 1983), interview concerning salivation (Baum & Bodner, 1983), electromyography (Hanawa et al., 2008), multidirectional lip‐closing force measurement system (Nakatsuka et al., 2011), and oral diadochokinesis (ODK) measuring the lip movement velocity (Ito et al., 2009). However, the objectivity of visual judgment and interviews is limited, and expert knowledge is necessary for evaluation using electromyography and the multidirectional lip‐closing force measurement system. Although ODK can easily evaluate the motor function of lips, it can only evaluate motor skills, which represent the agility of muscle movements.

A digital strain force gage (Lipple‐kun®) was recently developed as a device to objectively evaluate the muscle strength of the lips among the motor functions of the lips (Saitoh et al., 2017). This device enables a simple and accurate evaluation of lip‐seal strength, which is the muscle strength exerted by pursing of the lips (Ueda et al., 2019).

Lip‐seal strength and other motor functions of the lips appear to be related to oral motor functions such as pronunciation and mastication. However, there are few reports on lip‐seal strength in adults, with its variance and characteristics still unclear (Oki et al., 2021; Ueda et al., 2019). Therefore, lip‐seal strength in adults is currently utilized almost exclusively to evaluate changes in oral motor skills over time. Understanding the distribution of lip‐seal strength in adults and its characteristics will help in evaluating the lip‐seal strength of subjects and its interpretation. The null hypotheses of this study were (1) lip‐seal strength in adults is normally distributed and is not affected by age and (2) lip‐seal strength is not correlated with oral motor function. The objectives of this study were to understand (1) the distribution of lip‐seal strength in adult men and women as well as the effect of age on this strength and (2) identify if the oral motor function correlated with lip‐seal strength.

## 2 METHODS

### 2.1 Participants

The study subjects included outpatients who visited the Department of Prosthodontic Dentistry, Tokyo Dental College Suidobashi Hospital (Chiyoda city, Tokyo, Japan) and a community dental clinic (Edogawa city, Tokyo, Japan) for periodic examination and students from the Tokyo Dental College. The survey of this study was conducted from January 2015 to the end of May 2018. The inclusion criteria were the absence of objective and subjective abnormal movements and sensation of the lips. The exclusion criteria were the presence of tooth loss in the upper or lower anterior tooth regions that was left untreated without prosthesis, a markedly mobile tooth in the upper or lower anterior tooth regions, a muscle movement disorder around the oral cavity, oral or systemic acute symptoms, or those with reduced cognitive function judged as unable to properly understand the measurement method. In the case of measuring subjects wearing dentures, measurements were performed with the denture worn.

Oral function was evaluated by raters who received training in the usage of the devices and the evaluation method, and the methods were standardized before the trial.

### 2.2 Lip‐seal strength

Lip‐seal strength was measured using Lippule‐kun® (Shofu, Kyoto, Japan; Saitoh et al., 2017; Ueda et al., 2019; Oki et al., 2021) which has been reported to be a reliable measuring device (Ueda et al., 2019). Dental floss (Johnson & Johnson, New Jersey, USA), measuring 30‐cm‐long, was tied in a ring shape, and Lippule‐button® (Shofu, Kyoto, Japan) was attached to the tip of Lippule‐kun (Oki et al., 2021). Lippule‐kun has an oral screen‐like shape and was placed in the oral vestibule (Oki et al., 2021). During measurements, the subjects sat in a manner setting the head position parallel to the Frankfort plane and floor. They were also instructed beforehand about the following measurement conditions: resist traction using the muscle strength of the lips alone, do not resist traction using sucking force (do not make the intraoral pressure negative), and do not bend the head and body forward while resisting.

The measurer stood in front of the subjects and measured the lip‐seal strength. The subjects were instructed to close the lips after confirming that they had placed the Lippule‐button between the upper and lower anterior tooth regions, and the button was positioned at the center of dentition. Lippule‐kun was set at a position on the midline of the face and parallel to the floor to irradiate the subnasale with the indicator light. Then, the button was pulled slowly in the horizontal direction while maintaining the position of Lippule‐kun. The button was pulled until it was out of the lips, and the maximum value during this period was recorded using the Lippule‐kun. The subjects practiced this procedure several times before measurement. Measurement was performed after confirming that the subject had sufficiently understood the measurement method. Measurements were repeated three times while ensuring that a break was taken, and the maximum value was defined as the lip‐seal strength (N).

### 2.3 Other oral motor functions

For examining the relationship between lip‐seal strength and oral motor function, ODK representing the motor skill of the tongue and lips, tongue pressure representing the tongue muscle strength, occlusal force representing the chewing force of the upper and lower dentition, and masticatory performance representing the food‐crunching force were measured. ODK was evaluated using KENKOU‐KUN handy (Takei Scientific Instruments Co., Ltd., Niigata, Japan; Ito et al., 2009). The subjects were instructed to repeatedly pronounce /pa/, /ta/, and /ka/ for 5 s, respectively, as fast as possible, and the numbers of syllables pronounced per second were measured. The tongue pressure was evaluated using the JMS tongue pressure measurement device (JMS Co., Ltd., Hiroshima, Japan; Tsuga et al., 2011). The balloon of the tongue pressure probe was placed in the region anterior to the palate, and the subject pushed the balloon toward the palate with the tongue for 7 s. The tongue pressure was recorded three times, and the maximum value was adopted as the tongue pressure. The occlusal force was measured using Occlusal Force‐Meter GM10 (Nagano Keiki Co., Ltd, Tokyo, Japan; Hirao et al., 2014). The maximum occlusal force was measured at the bilateral first molars once each, and the maximum value was adopted as the occlusal force. The masticatory performance was measured using Gluco Sensor GS‐II (GC Corporation, Tokyo, Japan; Uesugi & Shiga, 2017). Subjects chewed gummi jelly containing glucose for 20 s, followed by holding 10 ml of distilled water in their mouth and spitting out all the materials held in the oral cavity into a cup with a filter. The gummi jelly pulverized by the filter was separated, and the glucose level in the filtrate was measured using Gluco Sensor GS‐II. The measured volume of the extracted glucose was considered as the masticatory performance.

### 2.4 Statistical analysis

**FIGURE 1:**
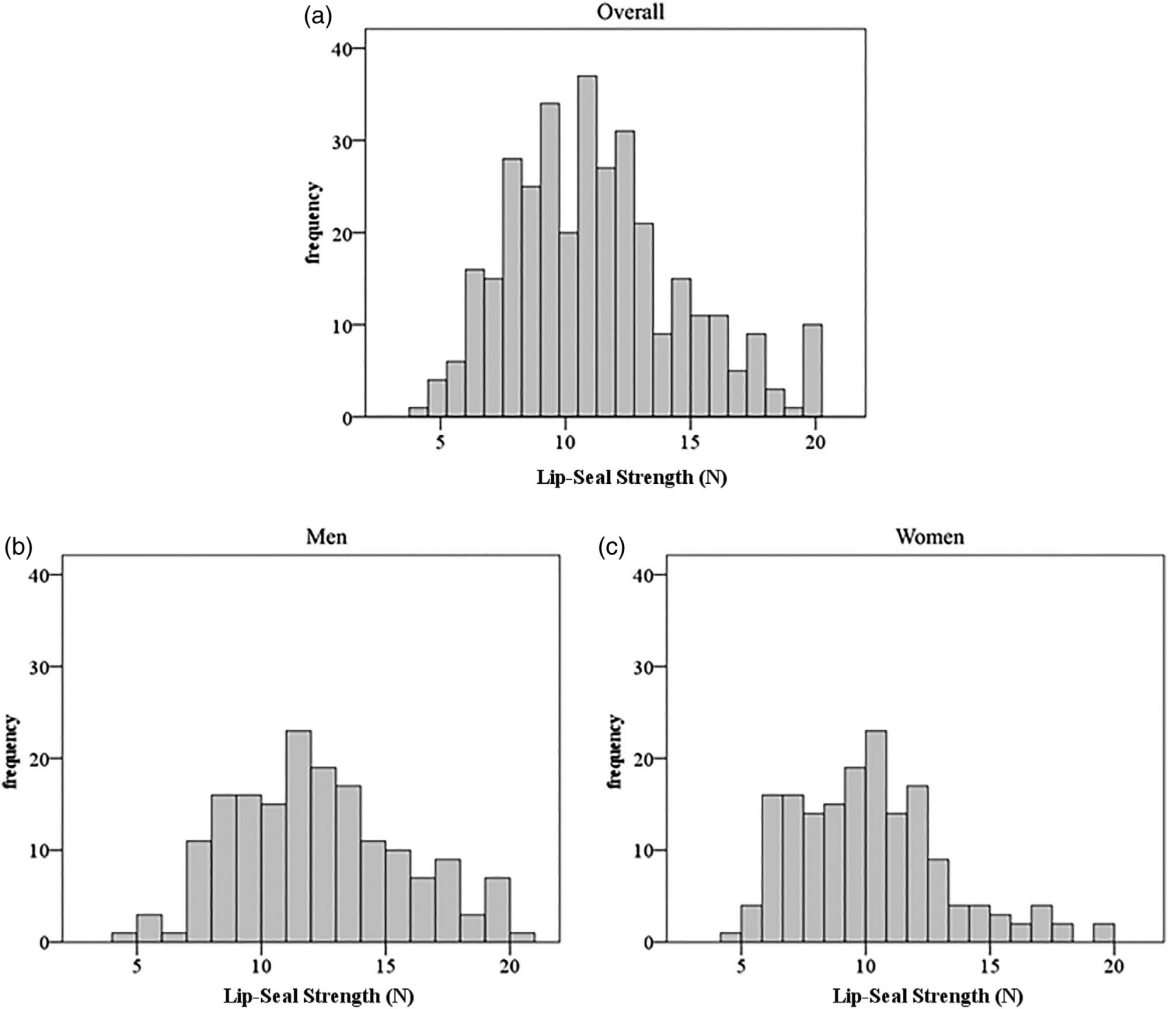
Histograms of lip‐seal strength (a) histograms of lip‐seal strength in all subjects, *n* = 339, (b) histograms of lip‐seal strength in men, *n* = 170, (c) histograms of lip‐seal strength in women, *n* = 169

Figure 1 shows the histograms of lip‐seal strength. The normality of the lip‐seal strength was examined using the Shapiro–Wilk test. The *p*‐value of lip‐seal strength was <0.001 for all subjects; it was 0.015 for men and <0.001 for women, thus negating normality. The statistical analysis was performed using nonparametric tests as there was no normality of the distribution of lip‐seal strength values. The Mann–Whitney *U* test was used to analyze the sex differences in the lip‐seal strength and the other measured oral motor function. The effect size of the Mann–Whitney *U* test was calculated from the z‐score and the number of subjects. Analysis of trends in lip‐seal strength according to age was conducted using the Jonckheere–Terpstra test. Spearman's correlation analysis was used to examine the correlation between lip‐seal strength and the study items. The relationship between lip‐seal strength and age was examined by performing curvilinear regression analysis using quadratic curves. All analyses were performed using the statistical software IBM SPSS version 24 (IBM corp., New York, United States of America), with the statistical significance level set at 0.05. Because this study is a secondary analysis of a previous survey, no sample size calculations were performed.

## 3 RESULTS

### 3.1 Characteristics of subjects

During the survey period, a total of 339 patients (man: 170, age 39.2 ± 18.2 years; women: 169, aged 43.1 ± 19.7 years) agreed to participate in this study. None of these 339 subjects met the abovementioned exclusion criteria. Table 1 shows the characteristics of the study subjects. The lip‐seal strength (mean ± standard deviation [SD]) was 11.2 ± 3.4 N in all subjects, 12.3 ± 3.4 N in men, and 10.2 ± 3.0 N in women. Men had higher lip‐seal strength than women, demonstrating a significant sex difference.

**TABLE 1:** Characteristics of study subjects

	Overall (*n* = 339)		Men (*n* = 170)		Women (*n* = 169)			
	Mean (SD)	Median (Q1, Q3)	Mean (SD)	Median (Q1, Q3)	Mean (SD)	Median (Q1, Q3)	*p*‐value	Effect size
Age (years)	41.1 (19)	34 (25, 53)	39.2 (18.2)	32 (25, 48)	43.1 (19.7)	35 (25, 58)	0.154	0.077
Lip‐seal strength (N)	11.2 (3.4)	10.9 (8.7, 13.2)	12.3 (3.4)	11.9 (9.4, 14.4)	10.2 (3.0)	9.9 (8.0, 12.0)	<0.001	0.313
ODK /pa/ (times/s)	6.3 (0.9)	6.4 (5.6, 6.9)	6.4 (0.9)	6.4 (5.8, 7.0)	6.1 (0.9)	6.1 (5.6, 6.8)	0.001	0.198
ODK /ta/ (times/s)	6.6 (1.1)	6.6 (5.8, 7.4)	6.7 (1.0)	6.8 (6, 7.4)	6.4 (1.1)	6.4 (5.6, 7.2)	0.022	0.135
ODK /ka/ (times/s)	6.1 (1.0)	6.2 (5.4, 6.8)	6.2 (1.0)	6.4 (5.4, 6.8)	6.0 (0.9)	6.0 (5.4, 6.6)	0.117	0.092
Tongue pressure (kPa)	38.1 (9.2)	37.9 (32.2, 43.1)	41.2 (9.8)	40.5 (35.7, 47.0)	35.1 (7.3)	35.3 (30.6, 40.0)	<0.001	0.328
Occlusal force (kN)	0.391 (0.222)	0.344 (0.210, 0.543)	0.462 (0.22)	0.468 (0.275, 0.629)	0.314 (0.198)	0.300 (0.155, 0.416)	<0.001	0.341
Masticatory performance (mg/dL)	193.9 (67.2)	193.0 (149.5, 226.0)	201.8 (67.8)	199.0 (160.0, 234.0)	185.7 (65.7)	180.0 (140.0, 222.0)	0.014	0.145

*Note*: No sex difference was noted in the age of the subjects. The lip‐seal strength was higher in men than in women, showing a significant difference.

Abbreviations: ODK, oral diadochokinesis; Q1, first quartile; Q3, third quartile; SD, standard deviation.

### 3.2 Lip‐seal strength according to age

As there was a sex difference in the lip‐seal strength, subsequent analyses were performed in each sex. The lip‐seal strength according to age is shown in Table 2. In men, there was no change in lip‐seal strength with age. However, women showed a slightly decreased lip‐seal strength with age.

**TABLE 2:** Lip‐seal strength according to age

Lip‐seal strength (N)	20–29 (O:130, M:70, F:60)		30–39 (O:73, M:42, F:31)		40–49 (O:40, M:20, F:20)		50–59 (O:30, M:10, F:20)		60–69 (O:24, M:10, F:14)		70–79 (O:23, M:10, F:13)		80–89 (O:16, M:6, F:10)		90–99 (O:3, M:2, F:1)		
	Mean (SD)	Median (Q1, Q3)	Mean (SD)	Median (Q1, Q3)	Mean (SD)	Median (Q1, Q3)	Mean (SD)	Median (Q1, Q3)	Mean (SD)	Median (Q1, Q3)	Mean (SD)	Median (Q1, Q3)	Mean (SD)	Median (Q1, Q3)	Mean (SD)	Median (Q1, Q3)	*p* value
Overall	10.8 (3.5)	10.4 (8.1, 13.2)	11.6 (2.9)	11.2 (9.5, 13)	12.4 (3.9)	11.7 (9.0, 15.7)	11.4 (3.2)	10.9 (9.3, 12.8)	11.7 (3.3)	11 (9.6, 13.4)	9.9 (3.3)	9.6 (7.5, 12.4)	10.8 (3.3)	10.6 (8, 12.4)	10.6 (3.2)	11.6 (−, −)	0.407
Men	12.1 (3.6)	12.5 (9.2, 14.3)	12.0 (3.0)	11.4 (9.4, 13.9)	13.7 (4.0)	14.1 (10.8, 17.4)	12.1 (3.2)	11.4 (9.6, 13.2)	12.3 (3.4)	11.7 (10.2, 15.4)	11.6 (3.3)	11.1 (8.9, 14.0)	11.6 (4.1)	10.2 (8.2, 16.1)	12.4 (1.1)	12.3 (−, −)	0.965
Women	9.2 (2.7)	8.7 (7.1, 10.7)	11.1 (2.7)	11 (9.5, 12.3)	11 (3.3)	10.4 (8.7, 12.4)	11.1 (3.2)	10.6 (8.8, 12.9)	11.3 (3.3)	10.8 (9.4, 13.2)	8.7 (2.9)	8.1 (6.6, 10.9)	10.4 (2.9)	10.6 (7.6, 12.3)	7.1 (0.0)	7.1 (−, −)	0.047

*Note*: The lip‐seal strength slightly decreased with age in women. In men, the lip‐seal strength did not decrease with age.

Abbreviations: Q1, first quartile; Q3, third quartile; SD, standard deviation.

### 3.3 Correlation between the lip‐seal strength and survey items

The results of Spearman's correlation analysis are presented in Table 3. Lip‐seal strength showed a significant correlation with sex. In addition, a significant positive correlation was observed between lip‐seal strength and age only in women. Lip‐seal strength was found to be significantly correlated with tongue pressure, occlusal force, and masticatory performance in men. In women, it showed a significant correlation with /pa/ and /ta/ of ODK, tongue pressure, and masticatory performance. The oral motor function, which showed a significant corelation with lip‐seal strength, differed between men and women.

**TABLE 3:** Correlation between lip‐seal strength and survey items

	Lip‐seal strength (N)					
	Overall		Man		Woman	
	*r*	*p*‐value	*r*	*p*‐value	*r*	*p*‐value
Age (years)	0.082	0.133	0.041	0.598	0.161	0.037
Sex (0: women, 1: men)	0.313	<0.001	–	–	–	–
ODK /pa/ (times/s)	0.206	<0.001	0.117	0.159	0.202	0.015
ODK /ta/ (times/s)	0.155	0.008	0.064	0.439	0.169	0.043
ODK /ka/ (times/s)	0.148	0.011	0.107	0.198	0.149	0.075
Tongue pressure (kPa)	0.335	<0.001	0.257	0.001	0.288	<0.001
Occlusal force (kN)	0.215	<0.001	0.195	0.017	0.068	0.428
Masticatory performance (mg/dL)	0.295	<0.001	0.204	0.012	0.315	<0.001

*Note*: The lip‐seal strength significantly correlated with age in women. In men, no significant correlation was noted between age and lip‐seal strength.

Abbreviation: ODK, oral diadochokinesis.

### 3.4 Relationship between lip‐seal strength and age

**FIGURE 2:**
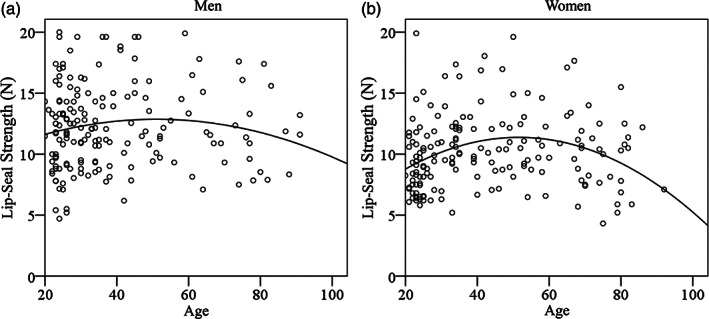
Relationship between lip‐seal strength and age (a) approximate curve between age and lip‐seal strength in men, (b) approximate curve between age and lip‐seal strength in women a significant association between age and lip‐seal strength in women was clarified. In men, no significant association was noted between age and lip‐seal strength

Figure 2 shows the approximate curves of lip‐seal strength and age acquired by the curvilinear regression analysis. The curvilinear regression analysis revealed *y* = −0.001*x* ^2^ + 0.131*x* + 9.504 (*R* ^2^ = 0.015, *p* = 0.289) in men, with no significant association between lip‐seal strength and age. In women, it was *y* = −0.003*x* ^2^ + 0.27*x* + 4.424 (*R* ^2^ = 0.084, *p* = 0.001), confirming a significant association between lip‐seal strength and age.

## 4 DISCUSSION

Decreased complex oral function has been suggested to worsen the general health (Hironaka et al., 2020; Tanaka et al., 2018). Reduced lip‐seal strength is also believed to be one of the factors causing a decline in the complex oral functions; however, reports on lip closure strength in adults are few (Oki et al., 2021; Ueda et al., 2019). Therefore, the variance and distribution of lip‐seal strength in adults is also not clear, and the target value for judging its decline has not yet been determined. Therefore, in this study, we attempted to characterize the lip‐seal strength of adults and provide information that can be part of the basis for determining the lip‐seal strength level of adults. This is the first study to measure the lip‐seal strength among adults from to a wide age range and examine the characteristics of lip‐seal strength.

Regarding the primary evaluation item in this study, the lip‐seal strength, intra‐ and inter‐rater reliabilities of measurement have been previously reported (Ueda et al., 2019). The lip‐seal strength was evaluated according to the measurement method (Ueda et al., 2019) with confirmed reliability and was measured by operators who standardized the measurement method. The measurement results of lip‐seal strength acquired by multiple raters were integrated and analyzed, and the results were found to be consistent. The devices used to measure lip‐seal strength, tongue pressure, occlusal force, and masticatory performance were those approved as inspection apparatuses for medical use in Japan.

The effect size of the Mann–Whitney *U* test for lip‐seal strength in this study was 0.313. Effect sizes serve only as a guide, and interpretations are known to vary depending on the research field and the statistical analysis used. Therefore, uniformly judging the effect size of all studies is not possible. In the field of speech, language, and hearing, which is considered as being relatively similar to the field of dentistry, Pearson r of 0.25, 0.40, and 0.65 were reported as small, medium, and large effect sizes, respectively (Gaeta & Brydges, 2020). Using this report as a guide, the effect size of the Mann–Whitney *U* test, which examined the sex difference in the lip‐seal strength in this study, was medium.

In the present study, a sex difference was noted in the lip‐seal strength. Such a difference in lip muscle strength has been previously reported (Miyamoto et al., 2019; Murakami et al., 2012), which was confirmed in the present study. Furthermore, the tongue pressure and the masticatory performance of oral motor function exhibited a significant correlation with lip‐seal strength in both sexes. Previous studies have also reported the association of the motor function of the lips with chewing (Ingervall, 1978) and tongue pressure (Hashiguchi et al., 2017). In these studies, the age group that the subjects belonged to and the devices used were different from those used in this study. However, the results of this study are similar to those of previous studies, which potentially supports the validity of our results.

The relationship between lip‐seal strength and age was investigated using the Jonckheere–Terpstra test, Spearman's correlation analysis, and curvilinear regression analysis. The results showed that there was no significant relationship between lip‐seal strength and age in men by all methods of analysis. It has been reported that ODK /pa/, which indicates the rapidity of lip movement, decreases in function with age (Watanabe et al., 2017; Watanabe et al., 2018). In addition, the orbicularis oris muscle strength and endurance, measured using an instrument different from that used in the present study, was reportedly lower in older subjects than in young adults (Park et al., 2018). These results differ from those of the present study. The orbicularis oris muscle is a complex multi‐layered muscle, regarded as a single muscle anatomically, but acts independently or together with other facial muscles functionally (Jain & Rathee, 2021). Lip‐seal strength, which represents the ability to resist the force of pulling the Lippule‐button in the closed mouth and to keep the Lippule‐button in the oral vestibule, is believed to be expressed by the interplay of several muscles, including the orbicularis oris muscle. The skeletal muscle fibers in the orbicularis oris muscle are reportedly composed of 22%–30% slow fibers, which contract slowly and are resistant to fatigue, and 66%–73% fast fibers, which contract rapidly (Hwang et al., 2007). In the tongue muscle, which moves in various ways akin to the orbicularis oris muscle, the composition of fast fibers reportedly decreases and that of slow fibers increases with age (Cullins & Connor, 2017). Assuming that the orbicularis oris muscle shows the same trend as the tongue muscle, this may potentially support our results in that the rapidity of lip movement decreases with age, whereas lip‐seal strength is not age‐related. Basic research has also shown that some muscles, such as the thyroarytenoid and the posterior cricoarytenoid, may be less sensitive to the effects of age (Nishida et al., 2013). Lip‐seal strength may be less affected by aging, but this study design could not explain the differences in the impact of aging on sex. The factors and specific mechanisms that lead to a decline in lip‐seal strength, which is a result of the coordination of multiple muscles, should be studied in more detail in the future.

The oral motor functions that correlated with lip‐seal strength were examined with respect to sex using Spearman's correlation analysis. Results showed no correlation of lip‐seal strength with any ODK item in men. In contrast, in women, the ODK /pa/ representing the motor skill of the lips and the ODK /ta/ representing the motor skill of the tongue tip correlated significantly with lip‐seal strength. However, in women, unlike in men, there was no correlation between lip‐seal strength and occlusal force. Thus, our results clarified that the oral motor functions related to lip‐seal strength differed between sexes.

This study has several limitations. Lip‐seal strength may be influenced by lateral craniofacial morphology (Doto & Yamada, 2015), dental arch morphology (Takehana et al., 2017), and the size of an attached removable denture (Schimmel, Memedi, et al., 2017). Therefore, it is important to consider the effects of these elements to appropriately evaluate the lip‐seal strength. However, it was not possible to examine the effects of these factors in this study. The findings of this study were similar to those of previous studies (Hashiguchi et al., 2017; Ingervall, 1978; Miyamoto et al., 2019; Murakami et al., 2012), suggesting that these factors had limited effects on the study results.

Regarding the subjects, we selected students and those patients who visited the outpatient services. All subjects came to the consultation room on their own, suggesting that their physical and cognitive functions were maintained to a certain level, even among elderly patients. However, there is no clear criterion to judge whether subject's movement and sensory function of the lips are normal or not. Hence, it may not be possible to conclude that the lip‐seal strength was normal based on these study results. Despite these limitations, this study was significant in reporting the lip‐seal strength in a wide range of age groups in the current state with no clarified lip‐seal strength value in adults. Therefore, using the results of this study as one guide, it may be possible to evaluate the lip‐seal strength level in adults.

Currently, as few reports are available on lip‐seal strength in adults, continued research is warranted (Oki et al., 2021; Ueda et al., 2019). One pressing issue is to determine a standard to measure the decline in lip closure strength in adults. Although there are several limitations in this study, we have suggested a simple guide for easy use in clinical practice based on our findings in a large number of adult subjects. We considered that if the value of lip‐seal strength in adults found in this study was lower than −1 SD or the first quartile, then decreased lip‐seal strength could be suspected. Applying this idea, the values were 8.9 N or 9.4 N in men and 7.2 N or 8.0 N in women, respectively. Based on these values, we suggest using 9.0 and 8.0 N for men and women, respectively, as a simple guide for screening for decreased lip‐seal strength.

Lip muscle strength can be improved by training (Fujiwara et al., 2016; Hägg et al., 2015; Hägglund et al., 2020; Kaede et al., 2016; Oki et al., 2021). Standardized lip strength training devices such as the Lipple Trainer® (SHOFU INC., Kyoto, Japan; Oki et al., 2021), IQoro® (MYoroface AB, Hudiksvall, Sweden; Hägg et al., 2015), and Muppy® (Dr. Hinz Dental, Herne, Germany; Hägglund et al., 2020) are currently available in the market. When the value is lower than the screening value or a reduction is noted in the measurement over time, the operator may propose training to maintain and improve lip‐seal strength. This may help to prevent communication disorder and reduction of oral motor functions such as chewing and swallowing caused by a decrease in lip‐seal strength.

The conclusions of this study were as follows: (1) lip‐seal strength was non‐normally distributed in both men and women, and lip‐seal strength was affected by age only in women; and (2) lip‐seal strength and multiple oral motor functions were significantly correlated, particularly tongue pressure and masticatory performance. The null hypotheses of this study were thus rejected. Because the indicators of perioral muscle strength and performance were correlated with lip‐seal strength, lip‐seal strength may also partially reflect the condition of the perioral muscles.

## CONFLICT OF INTEREST

The author declares that there is no conflict of interest.

## AUTHOR CONTRIBUTIONS

Yoshihiro Kugimiya, Kaoru Sakurai, and Takayuki Ueda designed the study and substantially contributed to the drafting of the manuscript. Takeshi Oki, Midori Ohta, and Masahiro Ryu substantially contributed analysis and interpretation of data. Kenichiro Kobayashi substantially contributed to acquisition of data. All authors contributed to the preparation and critical revision of the manuscript, and agree to be accountable for all aspects of the study.

## ETHICS STATEMENT

This study was approved by the ethics committee of the Tokyo Dental College (approval no. 755, 851) and conducted in accordance with the World Medical Association Declaration of Helsinki.

## Data Availability

The data that support the findings of this study are available from the corresponding author upon reasonable request.
